# Investigation of RhoA, ROCK1, and ROCK2 Gene Expressions in Autism Spectrum Disorders

**DOI:** 10.7759/cureus.74810

**Published:** 2024-11-30

**Authors:** E. Merve Kalınlı, Etem Akbas, Duygu Yolal Ertural, Serkan Gunes, Kansu Buyukafsar

**Affiliations:** 1 Department of Psychology, Toros University, Mersin, TUR; 2 Department of Medical Biology, Mersin University, Mersin, TUR; 3 Medical Biology, Independent Researcher, Mersin, TUR; 4 Child Psychiatry, Adana City Training and Research Hospital, Adana, TUR; 5 Pharmacology, Mersin University, Mersin, TUR

**Keywords:** autism spectrum disorder, rhoa, rho/rho-kinase signaling pathway, rock1, rock2

## Abstract

Objective: Autism Spectrum Disorder (ASD) is a neurodevelopmental condition that emerges in early childhood and is characterized by difficulties in social communication, repetitive behaviors, and restricted interests. The Ras homolog (Rho)/Rho-kinase signaling pathway plays a critical role in maintaining synaptic structure and function, as it regulates the actin cytoskeleton. This study aims to investigate the expression of the Ras homolog (Rho) family member A (*RhoA*), Rho-kinase 1 (*ROCK1*), and Rho-kinase 2 (*ROCK2*) genes within this pathway in relation to ASD.

Methods: The study included 82 individuals diagnosed with ASD from the Adıyaman Training and Research Hospital's Department of Child and Adolescent Psychiatry and 82 healthy individuals without a family history of ASD, matched for gender and age. RNA isolation and complementary DNA (cDNA) extraction for *RhoA*, *ROCK1*, and *ROCK2* genes were performed from blood samples of the patient and control groups. The *RhoA*, *ROCK1*, and *ROCK2* gene expression levels were analyzed by real-time polymerase chain reaction (PCR) using the comparative CT (ΔΔCT) method.

Results: Of the 82 individuals in the ASD group, 54 (65.9%) were male, and 28 (34.1%) were female, with a mean age of 7.74 ± 4.35 years. ASD is more common in males (p < 0.001). RhoA gene expression level is lower in patients with ASD (p < 0.001).

Conclusion: The Rho/Rho-kinase signaling pathway genes, including *RhoA*, *ROCK1*, and *ROCK2*, play roles in the neurodevelopmental processes. The lower expression level of RhoA in the ASD group may suggest that these genes could serve as potential biomarkers for the disorder. Further research is needed to explore these genetic markers' roles and their potential as therapeutic targets in ASD treatment.

## Introduction

Autism Spectrum Disorder (ASD) manifests in early childhood and is characterized by two main symptom groups. The first of these is the impairments in social communication and interaction (impairments in social-emotional response, impairments in non-verbal communication, difficulties in initiating, maintaining, and understanding relationships, etc.); the second is limited, repetitive interests and behaviors (repetitive motor movements, insistence on sameness, restricted areas of interest, increased or decreased sensory interests or reactions, etc.) [1]. The diagnosis of ASD is thought to have the presence of these clinical findings. Patients with ASD may have these findings in different combinations and at different severities. Personal differences between individuals with ASD are greater than seen in other psychiatric disorders [2].

According to data from the Autism and Developmental Disorders Surveillance Network of the Centers for Disease Control and Prevention (CDC), the prevalence of ASD was reported as 1/110 in 2006, 1/68 in 2010, and 1/54 in 2016. According to the last report of the same working group for 2018, published in 2021, this rate increased to 1/44 [3,4]. The incidence of ASD was found to be 3.5-4 times higher in males than in females [5].

Many environmental and genetic factors have been investigated about the etiology of ASD. It is accepted that there is a multifactorial, complex mechanism in ASD in which many genetic and environmental factors interact and combine in a common pathogenesis [6]. It is thought that disruptions in brain development, neuroimmune changes, neurotransmitter imbalances, gut-brain axis abnormalities, mitochondrial dysfunction, and irregularities in cell signaling pathways play a role in the pathogenesis of ASD [7]. Also, ASD is thought to be related to a pathology that concerns the entire brain and begins in infancy/early childhood, rather than a local disorder in a limited region of the brain [8]. It is emphasized in many articles that there is a disorganization in the number of connections between neurons and the structure of the synapses formed in individuals with ASD [9,10].

Previous studies show that the Rho/ROCK signaling system plays a critical role in many processes, such as the formation of the cytoskeleton, cell contraction, motility, and adhesion [11]. Cardiovascular [12], urogenital [13], gastrointestinal systems [14], and tumor pathogenesis [15] effects have been researched for many years, however, its effects on the central nervous system have attracted more attention in recent years and the information in this field is not yet sufficient.

Communication between neurons in the central nervous system occurs through synapses. During brain development, each neuron typically develops into an axon and numerous dendrites, forming neurites and, eventually, synapses. The ability to form precise neuronal connections depends on the spatial and temporal organization of multiple developmental steps, including axon specification, axon growth and branching, axon retraction/pruning, axon guidance and orientation, selection of synaptic target sites, dendritic growth and branching, and synapse formation [16]. Especially, the majority of excitatory synapses are located in protrusions on the dendrites that are enriched in the actin cytoskeleton. Experimental studies have shown that changes in the size and quantity of dendritic spines are largely dependent on actin cytoskeleton remodelling and are associated with synaptic transmission [17]. The Rho/ROCK pathway in the central nervous system has an important role in synaptic structure and function as it is a potent regulator of the actin skeleton [18]. Disorganization of any of the steps during the establishment of neuronal connections has significant effects on brain function and causes neurodevelopmental disorders like mental retardation [19], epilepsy [20], autism [21], and schizophrenia [22].

When the genetic etiology of ASD was investigated, it was shown in 2019 that more than 800 genes may be related to ASD. Most of these genes are related to synapse formation, maturation, and synaptic transmission. In more detail, 8.53% were associated with genes encoding Rho-guanine nucleotide exchange factors (Rho-GEFs), 12.28% with Rho-GTPase-activating proteins (GAPs), and 8.21% with Rho effectors [23]. The majority of genes associated with ASD and related to the Rho/ROCK pathway are expressed in the hippocampus, cortex, amygdala, and cerebellum in adult brains. These brain regions are related to social interaction and emotional responses, and brain imaging studies have shown different activation in these regions than normal in individuals with ASD [24]. In vivo studies with mice with knockouts for some of these genes (*Trio c* (Trio Rho guanine nucleotide exchange factor c), *Prex1* (Phosphatidylinositol-3,4,5-trisphosphate-dependent Rac exchange factor 1), *Dock4 *(Dedicator of cytokinesis 4), *Arhgef10* (Rho guanine nucleotide exchange factor 10), *Ophn1* (Oligophrenin 1), *Argap32* (Rho GTPase activating protein 32)) showed impaired social behavior and repetitive movements; and also mice homozygous for some genes such as *DOCK1* (Dedicator of cytokinesis 1), *ARHGEF10* (Rho guanine nucleotide exchange factor 10) and *NCKAP1* (NCK-associated protein 1) had severe developmental defects [23]. Although many genes belonging to Rho proteins have been associated with ASD, the role of these small G proteins and their downstream effectors (especially Rho-kinases) in the pathology has not yet been fully revealed. Considering the role of the Rho/ROCK pathway in actin cytoskeleton reorganization, cell membrane shape changes, neurite retraction, neuronal inflammation, and many other neuronal events, as well as the role of these events in ASD, it will be very interesting to investigate the contribution of the Rho/ROCK pathway in ASD [11].

## Materials and methods

Study design and subjects

This is a cross-sectional study conducted at Adıyaman Training and Research Hospital, Adıyaman, Turkey, between May 2020 and May 2021. The study was evaluated by the Mersin University Faculty of Medicine Ethics Committee and was found to be in accordance with decision number 2020/106 dated 05.02.2020.

Inclusion and exclusion criteria

The inclusion criteria of the patient group were 1) ASD diagnosis according to the Diagnostic and Statistical Manual of Mental Disorders, Fifth Edition (DSM-5) [1], 2) 0-18 years of age, 3) Not having a chronic physical illness. For the control group, the inclusion criteria were 1) No psychiatric disorder diagnosis according to DSM-5 [1], 2) 0-18 years of age, 3) normal intelligence capacity (IQ score above 70). Before the trial began, the guardians of all the participants provided written informed consent.

Study process

The study included 82 patients diagnosed with ASD and 82 healthy individuals who applied to the child psychiatry clinic with no family history of ASD at Adıyaman Education and Research Hospital. The diagnosis of autism and the mental health examination of the control group were made by an experienced child and adolescent psychiatrist. The study began with a consent statement in which participants' parents were assured that they agreed to participate in the study. Childhood Autism Rating Scale (CARS) was administered to all participants by an experienced child and adolescent psychiatrist. The principles of the Declaration of Helsinki were followed during the conduct of the study.

Sample size calculation

In order to say that the study is significant with 80% power and 5% Type 1 error, it is necessary to work with a minimum of 82 patients. When the literature studies were examined, Cohen’s d value for effect size was found to be 0.50 [25,26]. Accordingly, 82 healthy participants were deemed appropriate for this study. We followed a non-probability sampling technique (convenience sampling), as the sample was collected from easily accessible participants with no randomization.

Technique

Firstly, 6-7 ml of peripheral blood was taken from the individuals and placed in centrifuge tubes containing 1 ml of 2% ethylenediaminetetraacetic acid (EDTA). RNA isolation and cDNA synthesis were performed from these samples belonging to the patient and control groups, and then expression analysis was performed on these two sample groups. Trizol method was used to obtain RNA from peripheral blood. Trizol was added to the blood samples and incubated. After incubation, chloroform was added and centrifuged. Then isopropanol was added and centrifuged again. Finally, it was washed with ethanol. The RNA quality and quantity were assessed using a Nanodrop spectrophotometer (Thermo Fisher Scientific, Massachusetts, USA). The supernatant was discarded and dried, and the samples were stored at -20 degrees. Complementary DNA (cDNA) was synthesized using the High Capacity cDNA Reverse Transcription Kit (Applied Biosystems, Massachusetts, USA). The expression levels of *RhoA*, *ROCK1*, and *ROCK2* genes were analyzed using Real-time PCR with TaqMan probes (Applied Biosystems). The comparative CT (ΔΔCT) method was used for data analysis.

Statistical analysis

Statistical analyses were performed using SPSS software, version 22.0 (IBM Corp., Armonk, NY). Categorical data was presented by using descriptive statistics. Differences in gene expression levels between the ASD group and the control group were assessed using independent sample t-tests. Correlations between gene expression levels and clinical parameters were evaluated using Pearson correlation analysis. A p-value < 0.05 was considered statistically significant.

## Results

Demographic data

The ASD group consisted of 54 males (65.9%) and 28 females (34.1%), with a mean age of 7.91 ± 4.35 years. The control group consisted of 54 males (65.9%) and 28 females (34.1%), with a mean age of 8.08 ± 3.85 years. Control and ASD groups have similar age distribution in terms of gender and also ASD was more prevalent in males (p < 0.001) (Tables 1, 2).

**Table 1:** Distribution of demographic data ASD: Autism spectrum disorder.

Groups			N	%
ASD (n:82)	Gender	Male	54	65.9
		Female	28	34.1
	Socioeconomic Status	Low	25	30.5
		Medium	30	36.6
		High	27	32.9
	Family History of ASD	Yes	50	61.0
		No	32	39.0
Control (n:82)	Gender	Male	54	65.9
		Female	28	34.1
	Socioeconomic Status	Low	23	28.0
		Medium	34	41.5
		High	25	30.5
	Family History of ASD	Yes	0	0
		No	82	100.0

**Table 2:** Distribution of age and CARS score ASD: Autism spectrum disorder; CARS: Childhood autism rating scale.

	Groups	N	Mean±Sd	
Age	ASD	82	7.91±4.35	
	Control	82	8.08±3.85	
CARS score	ASD	82	37.56±3.17	
	Control	82	20.34±2.63	

Gene expression results

The expression levels of *RhoA* genes in the ASD group were significantly different from those in the control group (Table 3). When the *RhoA* gene expression level was examined according to gender within the groups, no statistically significant difference was detected between males and females in both groups.

**Table 3:** RhoA, ROCK1, ROCK2 gene expression levels in ASD and control groups ΔΔCT: Comparative CT (Cycle Threshold); ASD: Autism spectrum disorder.

	RhoA Expression (ΔΔCT)	p-value
ASD	1.361	< 0.001
Control	6.314	
	ROCK1 Expression (ΔΔCT)	0.061
ASD	3.648	
Control	2.166	
	ROCK2 Expression (ΔΔCT)	0.078
ASD	1.43	
Control	0.928	

The expression levels of *ROCK1 *and *ROCK2* genes in the ASD group were higher than those in the control group. However, this difference is not statistically significant (Table 3). When the *ROCK1* and *ROCK2* gene expression levels were examined according to gender within the groups, no statistically significant difference was detected between males and females in both groups.

## Discussion

The present study investigated the expression levels of *RhoA*, *ROCK1*, and *ROCK2 *genes in children with ASD compared to healthy controls. The primary findings reveal that the expression level of the *RhoA* gene is significantly lower in the ASD group, while the differences in the expression levels of *ROCK1* and *ROCK2* genes are not statistically significant. These findings may provide critical insights into the potential role of the Rho/Rho-kinase signaling pathway in the pathophysiology of ASD.

The significant reduction in RhoA gene expression in the ASD group suggests that RhoA may play a crucial role in the development and function of the central nervous system. RhoA is a member of the Rho family of small GTPases, which are known to regulate the actin cytoskeleton, essential for maintaining synaptic structure and function [27]. Previous studies have demonstrated that disruptions in RhoA activity can lead to impaired synaptic plasticity, which is a hallmark of several neurodevelopmental disorders, including ASD [28].

The lower expression levels of RhoA observed in this study may contribute to the synaptic abnormalities seen in individuals with ASD. Synaptic dysfunction is a well-documented feature of ASD, characterized by atypical synapse formation, maintenance, and plasticity [10]. The RhoA signaling pathway is involved in dendritic spine morphology, axon guidance, and synaptic stability. Therefore, the downregulation of RhoA could lead to altered neuronal connectivity and synaptic function, contributing to the core symptoms of ASD, such as deficits in social communication and repetitive behaviors.

Although the differences in *ROCK1 *and *ROCK2 *gene expressions between the ASD and control groups were not statistically significant, the trend toward higher expression levels in the ASD group warrants further investigation. *ROCK1* and *ROCK2* are downstream effectors of *RhoA* and play pivotal roles in regulating actin cytoskeleton dynamics, cell migration, and neurite outgrowth [11]. The observed increase in *ROCK1* and *ROCK2* expression levels in the ASD group, albeit not significant, might reflect compensatory mechanisms in response to the reduced *RhoA* expression.

*ROCK1* and *ROCK2* are serine/threonine kinases that phosphorylate various substrates involved in cytoskeletal organization. Dysregulation of ROCK activity has been implicated in several neurological disorders, including epilepsy, schizophrenia, and Alzheimer's disease [29]. In the context of ASD, altered *ROCK* activity could impact neuronal morphology and connectivity, potentially contributing to the pathophysiological features of the disorder.

The study also explored the expression levels of *RhoA*, *ROCK1*, and *ROCK2* genes according to gender within each group. The results indicated that *RhoA* expression levels were higher in males within the control group and higher in females within the ASD group. Similarly, *ROCK1* expression levels were higher in males within the control group and higher in females within the ASD group. *ROCK2* expression levels were higher in females in both the control and ASD groups. These gender-specific differences in gene expression might be linked to the higher prevalence and severity of ASD symptoms observed in males. The sample size is relatively small, and larger studies are needed to validate these findings.

Gender differences in ASD prevalence and severity are well-documented, with males being more frequently diagnosed with ASD and often exhibiting more severe symptoms. The differential expression of *RhoA*, *ROCK1*, and *ROCK2* genes between males and females might contribute to these observed gender differences in ASD. Further research is needed to elucidate the underlying mechanisms driving these gender-specific variations in gene expression and their impact on ASD pathophysiology.

Implications for therapeutic interventions

The findings of this study may suggest that targeting the Rho/Rho-kinase signaling pathway could hold therapeutic potential for ASD. Pharmacological agents that modulate *RhoA* activity or inhibit *ROCK1 *and *ROCK2* may be explored as potential treatments for ASD. For example, *ROCK* inhibitors have shown promise in preclinical studies for various neurodevelopmental and neurodegenerative disorders by promoting neuronal survival, axonal regeneration, and synaptic plasticity [30].

However, it is essential to consider the complexity of the Rho/Rho-kinase signaling pathway and its involvement in multiple cellular processes. Therapeutic interventions targeting this pathway must be carefully evaluated to avoid potential adverse effects on other physiological functions regulated by *RhoA*, *ROCK1*, and *ROCK2*.

Limitations and future directions

While this study provides valuable insights into the role of the Rho/Rho-kinase signaling pathway in ASD, several limitations should be acknowledged. The study is conducted in a single location (Adıyaman Training and Research Hospital) and the findings may not be generalizable to other populations or regions and further studies in diverse settings are needed to confirm the findings. The sample size is relatively small, and larger studies are needed to validate these findings. Additionally, the study focused on gene expression levels in peripheral blood samples, which may not fully represent the molecular changes occurring in the brain. Future studies should aim to analyze brain tissue samples or employ advanced imaging techniques to gain a more comprehensive understanding of the Rho/Rho-kinase pathway's involvement in ASD. Although controls were matched for age and gender, we did not assess other potential confounding factors (e.g., socioeconomic status, environmental factors, nutritional status, comorbid psychiatric conditions). Since these conditions may also affect gene expression and ASD symptoms, further studies are needed to evaluate these conditions additionally. This could be considered another limitation of the study.

Furthermore, this study did not investigate the functional consequences of altered gene expression on neuronal connectivity and behavior. Future research should incorporate functional assays and animal models to explore the impact of *RhoA*, *ROCK1*, and *ROCK2* dysregulation on neural circuits and ASD-related behaviors.

## Conclusions

In conclusion, this study highlights the potential involvement of the Rho/Rho-kinase signaling pathway in the pathophysiology of Autism Spectrum Disorder. The significant reduction in *RhoA* gene expression in individuals with ASD may suggest that *RhoA* could serve as a potential biomarker for the disorder. Although the differences in *ROCK1* and *ROCK2* gene expressions were not statistically significant, the observed trends indicate the need for further investigation. Understanding the molecular mechanisms underlying ASD can pave the way for the development of targeted therapies aimed at improving the lives of individuals with this complex neurodevelopmental disorder.
