# Correction: Investigation of RhoA, ROCK1, and ROCK2 Gene Expressions in Autism Spectrum Disorders

**DOI:** 10.7759/cureus.c206

**Published:** 2025-01-15

**Authors:** E. Merve Kalınlı, Etem Akbas, Duygu Yolal Ertural, Serkan Gunes, Kansu Buyukafsar

**Affiliations:** 1 Department of Psychology, Toros University, Mersin, TUR; 2 Department of Medical Biology, Mersin University, Mersin, TUR; 3 Medical Biology, Independent Researcher, Mersin, TUR; 4 Child Psychiatry, Adana City Training and Research Hospital, Adana, TUR; 5 Pharmacology, Mersin University, Mersin, TUR

This article has been corrected to include Dr. Kansu Buyukafsar as fifth author. Dr. Buyukafsar was erroneously omitted from the original author list despite extensive contributions to the work. All authors have agreed to this correction.

